# Weight loss technology for people with treated type 2 diabetes: a randomized controlled trial

**DOI:** 10.1186/s12986-017-0163-9

**Published:** 2017-01-31

**Authors:** Kuat Oshakbayev, Bibazhar Dukenbayeva, Gulnar Togizbayeva, Aigul Durmanova, Meruyert Gazaliyeva, Abdul Sabir, Aliya Issa, Alisher Idrisov

**Affiliations:** 1 Metabolic Syndrome Department, Nazarbayev University Medical Center, Street Baitursynuly, 5, fl. 601, 010000 Astana, Kazakhstan; 2 Faculty of Pathology and Forensic Medicine, Medical University Astana, Astana, Kazakhstan; 3 Faculty of Cardiology, Medical University Astana, Astana, Kazakhstan; 4 Department of Endocrinology, Republican Diagnostic Center at Nazarbayev University Medical Center, Astana, Kazakhstan; 5 Faculty of Internal Medicine, Karaganda State Medical University, Karaganda, Kazakhstan; 6 Neurodevelopmental Services, Richmond Healthcare Civic Centre, Twickenham, London UK; 7 Department of Endocrinology, National Scientific Cardiac Surgery Center, Astana, Kazakhstan; 8 Department of Endocrinology, Medical University Astana, Astana, Kazakhstan

**Keywords:** Type 2 diabetes, Weight loss, Restriction diet, Glycemic/lipid and hormone profiles, Lipid/protein oxidation

## Abstract

**Background:**

The prevalence of type 2 diabetes is increasing in worldwide despite the development of new treatment methods. Aim of the study was to evaluate a weight loss method on body composition, glycemic, lipid and hormone profiles, blood pressure and reactive oxygen species in people with treated type 2 diabetes.

**Methods:**

A 24-week open, prospective, randomized, controlled clinical trial including 272 adult patients with treated type 2 diabetes was performed. The patients were divided in two groups: Main group consisted of 208 patients who followed a method including a calorie restriction diet and optimal physical activity; Control included 64 patients who received conventional drug treatment with weight loss. Main Outcome Measures were weight loss, fasting glucose and 2-hour oral glucose tolerance test (OGTT), HbA1c. Secondary endpoints were blood pressure, lipid and insulin blood levels.

**Results:**

At 24 weeks, patients in Main weight lost between 8-18 kg (10–21%); their body mass index significantly decreased (-4.2 kg/m2) as well as their waist circumference (-13 cm) compared to Control. In Main weight loss was achieved fatty mass reduction. In Main fasting glucose and OGTT, HbA1c, blood pressure, reactive oxygen species decreased significantly, whereas hemoglobin levels and heel bone mineral density increased. In Main blood insulin levels decreased by 72.0%, cortisol levels decreased by 40.7%, while testosterone levels in men increased by 2.4 times from baseline. The application of the weight loss method led to a decrease in drug doses leading to their complete withdrawal.

**Conclusions:**

The results of this study show the beneficial role of a weight loss method in improving glycemic, lipid and hormone profiles, electrolyte and biochemical indices, blood pressure, reactive oxygen species and bone mineral density in patients with treated type 2 diabetes.

**Trial registration:**

ClinicalTrials.gov Identifier: NCT02503865. Retrospectively registered November 2015.

## Background

In 2015, an estimated 422 million people had diabetes worldwide [1], with T2D making up to about 90% of the cases [2]. Diabetes is a major cause of blindness, kidney failure, heart attacks, stroke and lower limb amputation, and affects 8.5% of the adult population with equal prevalence in both women and men [3]. Diabetes triples the risk of cardiovascular diseases and doubles the risk of death [4]. Prevalence of T2D is increasing worldwide despite the development of new drugs.

Obesity/overweight is a major risk factor for T2D [5, 6]. For instance, just 5% weight reduction improves glycemic control in people with T2D [1, 6]. Effectiveness and types of weight loss approches in people with T2D are increasingly reported in the literature. and many tested weight loss methods have been proved to be less effective [4–6]. A systematic review of weight loss interventions in people with T2D revealed that interventions including very low-calorie diets along with moderate physical activity produced the largest effect [7, 8]. Although numerous studies have attempted to identify the optimal mix of lifestyle behavior for people with T2D, the optimal level of caloric restriction in T2D is not well defined. Moreover many of them do not include taking of sodium and androgen-safety behavior during weight loss. We have obtained positive results in people with T2D including those who are insulin-dependent, using a weight loss method that we developed. *Objective:* to evaluate the effects of our weight loss method on glycemic, lipid and hormone profiles, blood pressure (BP), reactive oxygen species level (ROS) and body composition in people with treated T2D in a 24-week open, prospective, randomized, controlled clinical trial.

## Methods

### Participants

The study enrolled 296 adult people aged from 25 to 74 years with treated T2D. All the participants had hypertension. The patients were randomly recruited and divided in two groups: Main group (Experimental): non-pharmacological weight loss; Control group (Conventional): conventional drug therapy with weight loss medication.

Main group included 224 patients who were taking anti-diabetes drugs at least 3 years prior to first visit. Of these 224 patients, 7.1% dropped out prior to the study completion: 6 patients moved to live on another town, 8 patients refused treatment through the weight loss method and 2 patients were excluded due to noncompliance. Therefore 208 patients (109 women, mean age 47.7 ± 1.8 years) with T2D were followed up. All these 208 patients had abdominal obesity with waist circumference (WC) 99.2 ± 0.6 cm and body mass index (BMI) 29.6 ± 0.4 kg/m^2^. 152 patients were on insulin (24–72 units/day), 56 patients were treated with Metformin, Sulfonylurea, incretines [exenatide (Byetta, Bydureon), liraglutide (Victoza)], Pioglitazone in different combinations.

Control group included 72 patients; 11.1% of them dropped out prior to study completion (2 patients moved to another town, 5 patients refused treatment, and one was excluded due to noncompliance). Thus 64 patients (34 women, mean age 46.5 ± 2.1 years, WC 98.9 ± 0.8 cm, BMI 29.3 ± 0.9 kg/m^2^) were recruited and treated with conventional drug therapy including hypoglycemics (metformin 500–1500 mg/day, exenatide 5–10 μg/day), lipid lowering (atorvastatin 40 mg/day), antihypertensive (lisinopril 20 mg/day, calcium channel blockers referring to benzodiazepines 90 mg/day) and anti-inflammatory (acetylsalicylate acid up to 2 g/day and/or thienopyridine class antiplatelet agent 75 mg per day) drugs, and a gastrointestinal lipase inhibitor for obesity management (orlistat 360 mg/day orally within one hour of completing a meal). 41 of these 64 patients were on insuli (24–72 U/day).

The study was carried out in Kazakhstan between January 2003 and December 2014 at the Scientific Research Institute of Cardiology and Internal Diseases (Almaty) and at the Nazarbayev University Medical Center (Astana).

#### Inclusion criteria

1) written informed consent form; 2) diagnosis of T2D ≥ 3-year and treatment with glucose lowering therapy; 3) triglycerides > 1.7 mmol/l or cholesterol > 5.6 mmol/l or both); 4) body fat% > 21; 5) WC > 94.0 cm in men or > 80.0 cm in women; 6) ongoing treatment with antihypertensive treatment; 7) weight loss treatment for 24 weeks and follow-up ≥ 24 weeks.

#### Outcome measures

*Primary endpoints:* weight loss over 24 weeks, fasting blood glucose and 2-hour oral glucose tolerance test (OGTT), HbA1c. *Secondary endpoints* were systolic/diastolic BP, blood lipids, blood insulin levels.

#### Randomization

An independent statistician unconnected with clinical practice prepared randomization lists of patients with T2D using computer generated random numbers (SPSS for Windows v.21.0: An IBM Company, Armunk, NY).

### Analytical assessment

T2D was diagnosed according to the criteria reported in the WHO/IDF 2006 consultation [*WHO/IDF consultation*. Geneva, Switzerland, 2006]. Hypertension was diagnosed by BP readings and from medical records. Abdominal obesity was assessed by WC using the standards for Asians developed by the International Diabetes Federation (2005). Physical activity of patients was assessed by the number of steps measured by pedometers (Hoffmann-La Roche Ltd, Basel, Switzerland).

Anthropometrical indicators included age (years), weight (kg), BMI (kg/m^2^), WC (cm). Body composition parameters including fat mass (in % of total body weight and total kg), visceral fat rating (units), fat free mass (kg), total body water (% and kg), muscle mass (% and kg), bone mass (% and kg), metabolic age (years), basal metabolic rate (kcal/day), and bioimpedance (Ohms) were measured using a Tanita-SC330S Body Composition Analyzer (Tanita Corp., Tokyo, Japan). On the same blood samples, complete blood cell count, erythrocyte sedimentation rate, urea, creatinine, glucose, electrolytes, total cholesterol, triglycerides, total proteins with fractions, fibrinogen, bilirubin, hepatic enzyme levels and HbA1c [normal: < 39 mmol/mol (5.7%); prediabetes: 39 to 48 mmol/mol (5.7–6.5%), diabetes: ≥ 48 mmol/mol (6.5%)] were determined. Post oral glucose load insulin sensitivity was determined using the 2-hour OGTT with 75 g of glucose. Ultrasound images (GE Vivid 7 Ultrasound; GE Healthcare Worldwide USA, Michigan) were obtained, and heel bone mineral density (HBMD) was measured using a Lunar Achilles Express Ultrasound densitometry (GE Healthcare USA, Madison; at normal range 100.0 ± 15.0 Units). Sodium excretion was considered high when >150.0 mmol/L for both genders in 24-hour collected urine (normal 135–145 mmol/L).

*For ROS we measured* malondialdehyde (MDA, a marker of lipid peroxidation and degradation product of oxidized fatty acids), superoxide dismutase and catalase (antioxidant enzymes), and advanced oxidation protein products (AOPP, markers of protein oxidation). MDA (μmol/L) was measured using a thiobarbituric acid-reactive substance assay as described by Yagi and modified by Ohkawa [9].

Antioxidant enzymes were determined in erythrocyte hemolysates, in which hemoglobin concentration was assayed using Drabkin’s method. Superoxide dismutase was measured by the method of Misra and Fridrovich [10]. Catalase was measured at λ =240 nm at 25 °C; catalase activity was expressed in U/g and one unit is considered as the amount of the enzyme which decomposes 1 g of H_2_O_2_ in 1 min at 25 °C in pH =7.0. AOPP (μmol/L) were measured by adding 40 μL of plasma to a 190 μL of mixture of 81% phosphate buffer solution, 10% glacial acetic acid and 4% 1.16 mmol solution of KI, followed by 2 min absorbance at 340 nm on a plate reader Multiscan Ascent using a spectrophotometric method. Chloramine-T solution was used as calibrator [11].

#### Hormonal assays

Fasting serum insulin (nU/L) was determined by immunoassay (Immunotech Insulin Irma kit, Prague, Czech Republic). Hyperinsulinemia was considered >12.5 nU/L.

The Homeostasis Model Assessment insulin resistance indexes (HOMA-IR) was used as surrogate measure of insulin sensitivity as follows: HOMA-IR = ((fasting insulin in nU/L) × (fasting glucose in mmol/l)/22.5). Insulin resistance was considered if the index was > 2.

Fasting serum cortisol was determined using solid-phase chemiluminescent enzyme immunoassay (Cortisol-IFA-Best kit, Russia) on Sunrise Tecan-5082 (Austria, Gmbh) at 450 nm (limit of detection was 5 nmol/L; measurement ranges were 0–1200 nmol/L. normal ranges 150–660 nmol/L).

Fasting total serum testosterone was assayed using standard solid-phase radioimmunoassay (Biermann DPC, Germany) procedures with a limit of detection < 4 ng/dL, 290–1290 ng/dL were normal values.

The hormones were investigated three days after stopping the treatment.

#### Intervention

For weight loss in Main group we used the “Analimentary detoxication” (ANADET) weight loss method [12], including calorie restriction to 100–150 kcal/day with fat-free vegetables (tomatoes and cucumbers), salt intake limitation to 5–6 g/day, walking at least 8000 steps/day, and sexual self-restraint. The exercise was promoted to favor blood circulation and decrease metabolic intoxication. The weight loss method consisted of two courses of “treatment” that lasted 14–28 days each according to the principle “the slower the weight loss, the longer its duration”, with a two-week interruption between the courses. Then the patients followed a diet where they ate one meal a day without any food restriction. A combination of in-person conversations and telephone calls were conducted during the 24-week study period and a 24-week follow-up period. Weight loss results were assessed by Tanita-SC330S, BMI, WC, and skinfold thickness measurement method (cm) in abdominal and the back areas. The method maintains an achieved target weight loss for ≥ 24 weeks.

### Statistics

The two-side Student’s *t*-test and Odds ratios (ORs) with 95% Confidence intervals (CIs) were used. The study data were tested against the normal distribution and are presented in Tables as Mean ± Standard Error of the Mean (M ± SEM), and in Figures as Mean ± Standard Deviation (M ± SD). The formula for the SEM is the SD divided by the square root of the sample size. The correlation analysis (r) and an analysis of covariance (ANCOVA) model were used. *P*-value of < 0.05 was set as significant and < 0.0001 was set as highly significant. Statistical analysis was performed using SPSS ver.21.0 for Windows (SPSS: An IBM Company, Armunk, NY) and Microsoft Excel-2013.

## Results

**Fig. 1:**
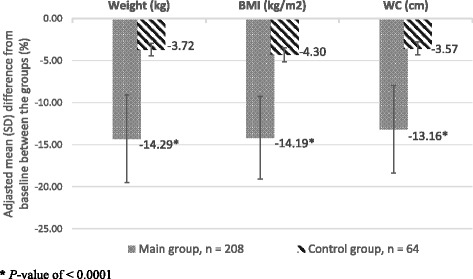
Adjusted mean differences in Anthropometrical data between baseline and week 24 in people with treated T2D in Main versus Control groups (SPSS, ANCOVA), *P* < 0.0001 (M ± SD)

**Fig. 2:**
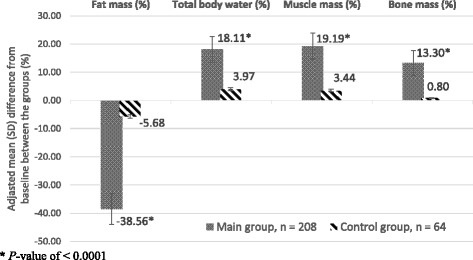
Adjusted mean differences in Body composition between baseline and week 24 in people with treated T2D in Main versus Control groups (SPSS, ANCOVA), *P* < 0.0001 (M ± SD)

Baseline/24-week treatment results concerning anthropometrical data, body composition, and metabolic data in the compared groups are shown in Table 1. In Main group weight loss varied from 8 to 18 kg (− 10–21% from baseline) and was higher than Control at 24-week (−12.8 ± 0.8 kg vs. -3.2 ± 0.9 kg, respectively; *P* < 0.00001). In Main we observed a significantly decrease in BMI (−4.2 kg/m^2^) and WC (−13 cm), skinfold thickness in abdomen and the back areas (−1.4 cm). Figure 1 shows adjusted mean differences in Main vs. Control from baseline in Anthropometrical data at 24-week. In Main weight loss was due to reduction of fat mass only (before, 25.41 ± 0.65 kg, and after, 12.38 ± 0.68 kg, *P* < 0.00001). Percentages of total body water and muscle masses tended to increase significantly, and the percentage of bone mass increased significantly too. Lean body mass (fat-free mass) did not change significantly during weight loss (*P* = 0.9225). Figure 2 shows adjusted mean difference from baseline Body composition in group Main vs Control at 24-week.

**Table 1:** Anthropometrical data, body composition, Metabolic age, Basal metabolic rate in people with treated T2D before (baseline) and after treatment (24-week) within Main (Experimental) and Control (Conventional) groups (M ± SEM)

Variables		Main group, *n* = 208		Control group, *n* = 64	
		Baseline	24-Week	Baseline	24-Week
Passport age (years)		47.69 ± 1.82		46.54 ± 2.08	
Weight (kg)		84.52 ± 0.75	72.44 ± 1.02**	84.49 ± 0.81	81.35 ± 1.23*
BMI (kg/m^2^)		29.60 ± 0.39	25.40 ± 0.37**	29.30 ± 0.47	28.04 ± 0.55
WC (cm)		99.18 ± 0.56	86.13 ± 0.93**	98.91 ± 0.83	95.38 ± 0.78*
Skinfold thickness in the areas of (cm):	Abdomen	3.96 ± 0.04	2.52 ± 0.05**	4.02 ± 0.07	3.69 ± 0.08*
	The back	4.41 ± 0.04	3.14 ± 0.05**	4.38 ± 0.06	4.06 ± 0.07*
Fat mass (%)		30.06 ± 0.52	18.47 ± 0.67**	29.73 ± 0.88	28.04 ± 0.92
Fat mass (kg)		25.41 ± 0.65	13.38 ± 0.68**	25.12 ± 0.97	22.87 ± 1.12
Fat free mass (kg)		59.11 ± 0.32	59.06 ± 0.41	59.37 ± 0.79	58.68 ± 0.80
Total body water (kg)		44.58 ± 0.26	45.13 ± 0.32	44.65 ± 0.44	44.81 ± 0.46
Total body water (%)		52.74 ± 0.35	62.29 ± 0.43**	52.84 ± 0.57	54.94 ± 0.62*
Muscle mass (kg)		54.22 ± 1.14	55.39 ± 1.96	54.21 ± 1.76	54.19 ± 1.98
Muscle mass (%)		64.15 ± 1.21	76.46 ± 1.62**	64.27 ± 1.71	66.48 ± 1.82
Bone mass (kg)		3.18 ± 0.06	3.09 ± 0.10	3.17 ± 0.09	3.07 ± 0.10
Bone mass (%)		3.76 ± 0.08	4.26 ± 0.09**	3.75 ± 0.09	3.78 ± 0.11
Metabolic age (years)		52.12 ± 1.52	42.31 ± 1.68**	52.01 ± 1.87	50.11 ± 1.92
Basal metabolic rate (kcal/day)		1702.4 ± 43.25	1401.6 ± 48.56**	1700.6 ± 50.45	1639.2 ± 55.44
Bioimpedance (ohms)		512.5 ± 9.25	465.5 ± 9.15*	507.4 ± 14.43	496.2 ± 13.28

*Abbreviations*: *BMI* body mass index, *M* mean, *SEM* standard error of the mean, *WC* waist circumference, *T2D* type 2 diabetes

*P-value of < 0.05, and

**P < 0.0001 were set as significant between baseline and 24-week treatment within group comparison

In Main group there was an inverse relationship between Fat mass percentage and Metabolic age/Basal metabolic rate (*r* = − 0.58, *P* = 0.0014 and *r* = − 0.68, *P* = 0.0009, respectively). Bioimpedance, electrical conductivity also improved.

The ANADET (Main group) led to a 15–19% decrease in systolic/diastolic BP in 199 patients (95.8%) (Table 2). Fasting Glucose, 2-hour OGTT, HbA1c and systolic BP decreased in Control, but to a lesser extent. In Control diastolic BP decreased less and did not reach the level recommended by the American Heart Association (2014).

**Table 2:** Blood pressures, Blood biochemical parameters, Lipid and protein oxidative products, Sodium excretion rate, and HBMD in people with treated T2D before (baseline) and after treatment (24-week) within the Main (Experimental) and Control (Conventional) groups (M ± SEM)

Variables	Main group, *n* = 208		Control group, *n* = 64	
	Baseline	24-week	Baseline	24-week
Systolic blood pressure (mmHg)	154.3 ± 1.22	120.4 ± 0.67**	150.4 ± 1.76	128.7 ± 2.17**
Diastolic blood pressure (mmHg)	98.7 ± 0.75	80.92 ± 0.63**	98.1 ± 0.98	93.2 ± 1.29*
Hemoglobin (g/L)	126.5 ± 0.68	138.9 ± 0.62**	127.1 ± 0.71	127.4 ± 0.72
ESR (mm/Hour)	23.4 ± 0.64	7.66 ± 0.26**	22.9 ± 0.70	24.8 ± 0.75*
Eosinophilia (%)	5.35 ± 0.21	1.82 ± 0.14**	5.29 ± 0.29	6.08 ± 0.30*
Glucose (mmol/L)	8.64 ± 0.19	4.50 ± 0.11**	8.58 ± 0.24	6.04 ± 0.21**
2-hour OGTT (mmol/L)	14.5 ± 0.28	6.7 ± 0.19**	14.1 ± 0.33	8.21 ± 0.42**
HbA1c (mmol/mol and %)	52.0 ± 0.68 (6.91 ± 0.09)	35.0 ± 0.52** (5.39 ± 0.08)	52.0 ± 1.13 (6.89 ± 0.15)	46.0 ± 1.15* (6.38 ± 0.16)
Cholesterol (mmol/L)	5.82 ± 0.07	4.54 ± 0.05**	5.77 ± 0.09	5.47 ± 0.11*
Triglyceride (g/L)	2.42 ± 0.08	0.81 ± 0.05**	2.21 ± 0.08	1.92 ± 0.12*
Total Fibrinogen (g/L)	4.52 ± 0.07	2.21 ± 0.06**	4.58 ± 0.09	4.05 ± 0.18*
MDA (μmol/L)	48.47 ± 0.68	33.48 ± 0.29**	46.99 ± 0.75	50.46 ± 0.79*
SOD (U/mg)	53.83 ± 0.54	77.71 ± 0.67**	52.91 ± 0.65	56.87 ± 0.97*
Catalase (U/g)	22.17 ± 0.42	26.44 ± 0.46**	23.81 ± 0.49	25.17 ± 0.68
AOPP (μmol/L)	266.8 ± 12.6	181.8 ± 7.8**	262.4 ± 13.6	295.6 ± 13.9*
Sodium in the urine (mmol/L)	164.1 ± 2.8	32.4 ± 1.9**	164.1 ± 2.8	182.4 ± 3.5**
Sodium in the blood (mmol/L)	134.4 ± 0.32	143.7 ± 0.38**	135.1 ± 0.35	134.1 ± 0.34*
HBMD (Units)	77.83 ± 1.86	93.2 ± 1.37**	78.78 ± 1.92	76.87 ± 1.88

*Abbreviations*: *ANADET* analimentary detoxication, *AOPP* advanced oxidation protein products, *ESR* erythrocyte sedimentation rate, *HBMD* heel bone mineral density, *M* mean, *MDA* malondialdehyde, *OGTT* oral glucose tolerance test, *SOD* superoxide dismutase, *SEM* standard error of the mean, *T2D* type 2 diabetes

*P-value of < 0.05, and

**P < 0.0001 were set as significant between baseline and 24-week treatment within group comparison

Hemoglobin, Catalase and HBMD levels did not change in Control, whereas in Main group hemoglobin levels increased by +9.5% and erythrocyte sedimentation rate and eosinophilia decreased from baseline by 67.5 and 66.0%, respectively. In Main group there was a reduction of fasting glucose (−47.9%), OGTT (2.2-fold), HbA1c (−22%), Cholesterol (−22.0%), Triglycerides (−66.5%), total Fibrinogen (−51.1%) (*P* < 0.0001). In Control HbA1c decreased by 8% (*P* < 0.05), but did not reach to the normal.

The oxidative products of lipids and proteins were also normalized in Main group, where MDA decreased by 30.9%, AOPP by 31.9%, and Superoxide dismutase increased by 44.4% and Catalase by 19.3% from baseline. Urine sodium excretion decreased by 80.3% after weight loss, while blood sodium increased by 6.7%. HBMD increased significantly (+19.7%) from baseline (*P* < 0.00001). In contrast, in Control there were a significant increase in eosinophilia, MDA, AOPP, and urine sodium and s decrease in blood sodium.

In Main after treatment blood insulin decreased by 72.0%, cortisol decreased by 40.7%, whereas blood testosterone in men (*n* = 99) increased 2.4-fold from baseline (Table 3). In Control, the positive changes in hormones were not so evident. For instance, insulin in Main decreased 3.6-fold from baseline after treatment, whereas in Control it decreased only 1.4-fold (95%; OR 2.6, CI 2.1–3.1). Although in Control insulin levels did not decrease to the normal. In addition, cortisol and testosterone levels did not change significantly in Control.

**Table 3:** Immunoassays of blood Insulin, HOMA-IR, Cortisol and Testosterone (in men) in people with treated T2D before (baseline) and after treatment (24-week) within Main (Experimental) and Control (Conventional) groups (M ± SEM)

Variables	Main group, *n* = 208		Control group, *n* = 64	
	Baseline	24-week	Baseline	24-week
Immunoassay insulin (nU/L)	23.2 ± 1.0	6.5 ± 0.3*	20.9 ± 1.2	15.0 ± 1.2*
Immunoassay cortisol (nmol/L)	751.2 ± 6.7	445.7 ± 5.3*	747.2 ± 8.9	739.5 ± 9.1
HOMA-IR index	8.9 ± 0.44	1.30 ± 0.29*	7.97 ± 0.53	4.03 ± 0.55*
Variable in men				
	Main group, *n* = 99		Control group, *n* = 30	
Blood testosterone (ng/dL)	365.9 ± 13.8	885.2 ± 21.2*	379.2 ± 19.6	395.2 ± 24.5

*Abbreviations*: *ANADET* analimentary detoxication, *HOMA-IR* Homeostasis Model Assessment insulin resistance index, *IR* insulin resistance, *M* mean, *SEM* standard error of the mean, *T2D* type 2 diabetes

*P < 0.0001 were set as significant between baseline and 24-week treatment within group comparison

**Fig. 3:**
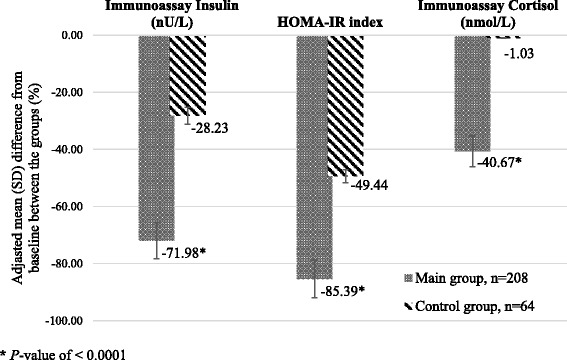
Adjusted mean differences in Immunoassays Insulin, Cortisol and HOMA-IR between baseline and week 24 in people with treated T2D in Main versus Control groups (SPSS, ANCOVA), *P* < 0.0001 (M ± SD)

The Table 3 proves that in Main group tissue insulin sensitivity improved following treatment, and IR (HOMA-IR index) decreased significantly 6.8-fold from baseline, whereas in Control it only decreased only 2-fold (95%; OR3.2, CI2.8–3.3). In Control the HOMA-IR index did not reach normal values (Fig. 3).

During the first 2–5 days of treatment most of the patients in Main complained of hunger, slight dizziness, weakness, lower extremity and abdominal muscle tremor, warm feeling in the umbilical/solar plexus areas, and psychogenic fear due to changed eating behavior, which all disappeared in the following days. 3–5 days after the start of the ANADET patients’ urine became turbid, muddy and intensively colored (dark). These urine appearance persisted for several days. Urine microscopy showed organic salts such as oxalates, urates, phosphates, and carbonates of calcium and magnesium. An increase in body temperature and blood leukocyte count was observed between 4 and 14 days after the start of ANADET. The comorbidity symptoms were also regressed gradually. The patients noticed a physical relief, increase in physical/mental workability, and exercise tolerance.

As the clinical status of the patients was improving, the previous anti-diabetic, antihypertensive and other symptomatic conventional medications were adequately decreased from 2 to 3 days after starting ANADET. By days 7 to 10 after the treatment start, the drugs were stopped completely. There was no recurrence of T2D at 6 months follow-up period if the patients had not regained overweight. If a patient regained overweight, symptoms of T2D gradually manifested again, and once again disappeared once the overweight was lost again.

## Discussion

Our data show that weight loss method (10 to 21% fat loss) including calorie restriction (100–150 kcal/day), fat-free vegetables and salt intake (5–6 g/day), optimal physical activity, and sexual self-restraint over a 24-week period is a safe, well tolerated, and acceptable nutritional therapy option for subjects with T2D. Our study confirms that weight loss can lead to normalization of BP [13]. ANADET repealed insulin resistance, improved blood insulin levels, increased hemoglobin, and improved inflammation parameters. Our results confirm those of some other studies [7, 8, 14]. ANADET also decreased blood cholesterol/triglyceride levels and lipid and protein oxidative products, and increased enzymes against oxidative stress. Our study prove a potential validity of the optimal mix of caloric restriction, walking, sodium intake and sexual self-restraint behavior in effective treatment of T2D.

People with T2D eliminate through urine more sodium ions due to hyperglycemia-induced serum osmolality [15]. Blood and urine sodium became normal on a weight loss method that decreased metabolic pollutants [16], with elimination of metabolites [17]. The sodium intake in Main group subjects with T2D helped to eliminate metabolic pollutants from the body.

ANADET method improved HBMD; notably, not all weight loss methods increase HBMD [18].

People with T2D have high blood cortisol [19] and low testosterone [20]. ANADET method improved both blood cortisol and testosterone levels. Numerous studies involving both animal and human models indicate that diet-induced weight loss is associated with positive changes in leptin and ghrelin levels, insulin sensitivity and cytokine patterns [21]. However, the reciprocal influences between adiposity and hormones like insulin, leptin, ghrelin and others remain unclear.

Overweight is a biological burden for the body consuming additional trophic, immunological, antitoxic, excretion function [22]. The ANADET method metabolizes of “old lipids”, leading to loss of fat mass only and to increase in muscle, water and bone masses. The patients experienced side effects related to symptoms of metabolic intoxication that is a common problem during weight loss. Adipose tissue can indeed absorb different persistent organic pollutants [23], and important role of adiposity in storing of pollutants has been outlined [24, 25]. ANADET method allows to: 1) control the elimination of metabolic end-products; 2) switch on lipolysis; 3) reuse of interim metabolites. Physical exercise and mandatory salt intake are important to decrease in metabolic wastes. If sodium ions are a main transporter in the body, then exercise improves the endothelial nitric oxide and prostaglandins pathways [26]. Sexual self-restraint allows to save endogenous androgens in both genders [27, 28].

The ability to accumulate adipose tissue is one of the most important adaptive mechanism for survival [29]. However, we are now observing a steady increase in obesity and related diseases [1–4].

### Study limitation

Published studies about positive role of weight loss in people with T2D are limited in scope and number. A limitation of our study is short duration. We acknowledge the clinical trial had approximately 8% of the randomly assigned population dropped out prior to completion. We should perform a multicenter prospective clinical randomized controlled trial. Further research is needed.

## Conclusions

In people with T2D an increased in fat mass is paralleled by reduction in muscle, bone, and water masses. Increased fat mass leads to increase bioimpedance and metabolic aging. Our results indicate that the weight loss method called ANADET can be an effective treatment for people with T2D. The method, aimed to cure and control metabolic intoxication, improves glycaemia, insulin resistance, lipids and hormones, electrolyte and biochemical outcomes, reactive oxygen species, instrumental parameter, and allows drugs reduction up to its complete withdrawal.

## Abbreviations

ANADET: Analimentary detoxication
AOPP: Advanced oxidation protein products
BMI: Body mass index
CI: Confidence intervals
ESR: Erythrocyte sedimentation rate
HbA1c: Glycosylated hemoglobin A1c
HBMD: Heel bone mineral density
HOMA: Homeostasis Model Assessment
IDF: International Diabetes Federation
IR: Insulin resistance
M: Mean
MDA: Malondialdehyde
OGTT: Oral glucose tolerance test
OR: Odds ratios
ROS: Reactive oxygen species
SD: Standard deviation
SEM: Standard error of the mean
SOD: Superoxide dismutase
T2D: Type 2 diabetes mellitus
WC: Waist circumference
WHO: World Health Organization

## References

[1] Global report on diabetes. World Health Organization, Geneva, 2016. http://apps.who.int/iris/bitstream/10665/204871/1/9789241565257_eng.pdf. Accessed 7 Apr 2016.

[2] Shi YH, Frank FB (2014). The global implications of diabetes and cancer. Lancet.

[3] Vos T, Flaxman AD, Naghavi M, Lozano R, Michaud C, Ezzati M (2012). Years lived with disability for 1160 sequelae of 289 diseases and injuries 1990–2010: a systematic analysis for the Global Burden of Disease Study 2010. Lancet.

[4] Jansson SP, Svardsudd K, Andersson DK (2014). Effects of fasting blood glucose levels and blood pressure and treatment of diabetes and hypertension on the incidence of cardiovascular disease: a study of 740 patients with incident Type 2 diabetes with up to 30 years’ follow-up. Diabet Med.

[5] Guariguata L, Whiting DR, Hambleton I, Beagley J, Linnenkamp U, Shaw JE (2014). Global estimates of diabetes prevalence for 2013 and projections for 2035. Diabetes Res Clin Pract.

[6] Wolf AM, Conaway MR, Crowther JQ, Hazen KY, Nadler J, Oneida B (2004). Improving Control with Activity and Nutrition (ICAN) Study. Translating lifestyle intervention to practice in obese patients with type 2 diabetes: Improving Control with Activity and Nutrition (ICAN) study. Diabetes Care.

[7] Snel M, Gastaldelli A, Ouwens DM, Hesselink MK, Schaart G, Buzzigoli E (2012). Effects of adding exercise to a 16-week very low-calorie diet in obese, insulin-dependent type 2 diabetes mellitus patients. J Clin Endocrinol Metab.

[8] Goday A, Bellido D, Sajoux I, Crujeiras AB, Burguera B, García-Luna PP (2016). Short-term safety, tolerability and efficacy of a very low-calorie-ketogenic diet interventional weight loss program versus hypocaloric diet in patients with type 2 diabetes mellitus. Nutr Diabetes.

[9] Ohkawa H, Ohishi N, Yagi K (1979). Assay for lipid peroxides in animal tissues by thiobarbituric acid reaction. Anal Biochem.

[10] Misra H, Fridrovich I (1972). The role of superoxide anion in the autooxidation of epinephrine and a simple assay for superoxide dismutase. J Biol Chem.

[11] Witko-Sarsat V, Friedlander M, Capeillere-Blandin C, Nguyen-Khoa T, Nguyen AT, Zingraff J (1996). Advanced oxidation protein products as a novel marker of oxidative stress in uremia. Kidney Int.

[12] Oshakbayev KP, Abylaiuly Z (2007). Clinical management of metabolic syndrome. Practice guidance.

[13] Jensen MD, Ryan DH, Apovian CM, Ard JD, Comuzzie AG, Donato KA (2014). 2013 AHA/ACC/TOS Guideline for the Management of Overweight and Obesity in Adults: A Report of the American College of Cardiology/American Heart Association Task Force on Practice Guidelines and The Obesity Society. J Am Coll Cardiol.

[14] Franz MJ, Boucher JL, Rutten-Ramos S, VanWormer JJ. Lifestyle weight-loss intervention outcomes in overweight and obese adults with type 2 diabetes: a systematic review and meta-analysis of randomized clinical trials. J Acad Nutr Diet. 2015;115(9):1447–63. 10.1016/j.jand.2015.02.031 25935570

[15] Liamis G, Liberopoulos E, Barkas F, Elisaf M (2014). Diabetes mellitus and electrolyte disorders. World J Clin Cases.

[16] Lee Y, Kim K, Kim S, Hong N, Lee S, Lee D (2014). Prospective associations between persistent organic pollutants and metabolic syndrome: A nested case–control study. Sci Total Environ.

[17] Lemke H, Carver T, Court O, Andersen R (2013). The impact of excess weight loss on bone mineral density ten years following bariatric surgery. Can J Diabetes.

[18] Chiodini I, Adda G, Scillitani A, Coletti F, Morelli V, Di Lembo S (2007). Cortisol secretion in patients with type 2 diabetes: relationship with chronic complications. Diabetes Care.

[19] Wang C, Jackson G, Jones TH, Matsumoto AM, Nehra A, Perelman MA (2011). Low testosterone associated with obesity and the metabolic syndrome contributes to sexual dysfunction and cardiovascular disease risk in men with type 2 diabetes. Diabetes Care.

[20] Gavrieli A, Mantzoros CS (2016). Novel molecules regulating energy homeostasis: physiology and regulation by macronutrient intake and weight loss. Endocrinol Metab (Seoul).

[21] Bluher M (2009). Adipose tissue dysfunction in obesity. Exp Clin Endocrinol Diabetes.

[22] Dirinck EL, Dirtu AC, Govindan M, Covaci A, Van Gaal LF, Jorens PG (2014). Exposure to persistent organic pollutants: relationship with abnormal glucose metabolism and visceral adiposity. Diabetes Care.

[23] Pestana D, Faria G, Sa C, Fernandes VC, Teixeira D, Norberto S (2014). Persistent organic pollutant levels in human visceral and subcutaneous adipose tissue in obese individuals-depot differences and dysmetabolism implications. Environ Res.

[24] Lee D-H, Porta M, Jacobs Jr DR, Vandenberg LN (2014). Chlorinated persistent organic pollutants, obesity, and type 2 diabetes. Endocr Rev.

[25] Bruder-Nascimento T, Silva ST, Boer PA, Cordellini S (2015). Effects of exercise training on stress-induced vascular reactivity alterations: role of nitric oxide and prostanoids. Braz J Phys Ther.

[26] Jiang M, Xin J, Zou Q, Shen JW (2003). A research on the relationship between ejaculation and serum testosterone level in men. J Zhejiang Univ Sci.

[27] Hamilton LD, Meston CM (2010). The effects of partner togetherness on salivary testosterone in women in long distance relationships. Horm Behav.

[28] Saltiel AR (2012). Insulin resistance in the defense against obesity. Cell Metab.

[29] Neeland IJ, Ayers CR, Rohatgi AK, Turer AT, Berry JD, Das SR (2013). Associations of visceral and abdominal subcutaneous adipose tissue with markers of cardiac and metabolic risk in obese adults. Obesity (Silver Spring).

